# Past and new challenges for malaria control and elimination: the role of operational research for innovation in designing interventions

**DOI:** 10.1186/s12936-015-0802-4

**Published:** 2015-07-17

**Authors:** Philippe Guyant, Vincent Corbel, Philippe J Guérin, Adeline Lautissier, François Nosten, Sébastien Boyer, Marc Coosemans, Arjen M Dondorp, Véronique Sinou, Shunmay Yeung, Nicholas White

**Affiliations:** Partners for Development, Phnom Penh, Cambodia; Institut de Recherche pour le Développement (IRD), Maladies Infectieuses et Vecteurs, Ecologie, Génétique, Evolution et Contrôle (IRD 224-CNRS 5290 UM1-UM2), Montpellier Cedex 5, France; Department of Entomology, Faculty of Agriculture, Kasetsart University, Bangkok, Thailand; Worldwide Antimalarial Resistance Network, Oxford, UK; Nuffield Department of Medicine, Centre for Tropical Medicine and Global Health, Oxford University, Oxford, UK; Initiative 5%, Expertise France, Paris, France; Shoklo Malaria Research Unit, Mahidol-Oxford Tropical Medicine Research Unit, Faculty of Tropical Medicine, Mahidol University, Mae Sot, Thailand; Medical entomology unit, Institut Pasteur de Madagascar, Tananarive, Madagascar; Institute of Tropical Medicine, Antwerp, Belgium; University of Antwerp, Antwerp, Belgium; Faculty of Tropical Medicine, Mahidol University, Bangkok, Thailand; Laboratory of Parasitology, Faculty of pharmacy, UMR-MD3, Aix-Marseille University, Marseille, France; Department of Global Health and Development, Malaria Centre, London School of Hygiene and Tropical Medicine, London, UK

## Abstract

This meeting report presents the outcomes of a workshop held in Bangkok on December 1st 2014, where the following challenges were discussed: the threat of resistance to artemisinin and artemisinin-based combination therapy in the Greater Mekong Sub-region (GMS) and in Africa; access to treatment for most at risk and hard to reach population; insecticide resistance, residual and outdoors transmission. The role of operational research and the interactions between research institutions, National Malaria Control Programmes, Civil Society Organizations, and of financial and technical partners to address those challenges and to accelerate translation of research into policies and programmes were debated. The threat and the emergency of the artemisinin resistance spread and independent emergence in the GMS was intensely debated as it is now close to the border of India. The need for key messages, based on scientific evidence and information available and disseminated without delay, was highlighted as crucial for an effective and urgent response.

**Electronic supplementary material:**

The online version of this article (doi:10.1186/s12936-015-0802-4) contains supplementary material, which is available to authorized users.

## Background

### Malaria situation worldwide

In over a decade, remarkable achievements in malaria control have been made. Malaria mortality rates have been reduced by 47% in all age groups worldwide, between 2000 and 2013, leading to an estimated 4.3 million malaria deaths averted during the same period. A total of 64 countries that had on-going malaria transmission in 2000 are actually meeting the millennium development goals (MDG) target of reversing the incidence of malaria, and 55 of these countries are on track to meet roll back malaria (RBM) and World Health Assembly targets of reducing malaria case incidence rates by 75% in 2015 [1].

In the last decade, resistance to commonly used insecticides and anti-malarial drugs has been emerging or expending and poses a major threat to the sustainability of these progresses. Monitoring of the resistance trends at country level is crucial to properly quantify and address those threats. In the last few years, new challenges have emerged, further complicating the potential solutions to the issue of drug and of insecticide resistance and to attain the goal of elimination.

### Objectives of the workshop

As a side event to the Roll Back Malaria Board Meeting, this 1 day workshop was organized by Expertise France (formerly known as France Expertise Internationale) in the framework of the 5% Initiative in Bangkok on December 1st 2014. This 5% Initiative is an indirect contribution from France to the Global Fund equivalent to 5% of the total French contribution to the Fund each year. It provides technical support to countries allocated Global Fund grants in designing, implementing, monitoring/evaluating and measuring the impact of programmes financed by the Global Fund in order to enhance their effectiveness and impact on health. The workshop convened a global community of over 80 researchers, representatives of National Malaria Control Programmes (NMCPs), of Civil Society Organizations (CSOs), and of financial and technical partners (list of participants as Additional file 1). The objectives of this workshop were:

- To discuss the new challenges for malaria control and elimination.
- To discuss the role of operational research for innovation in designing interventions.
- To share the results of projects supported by the 5% Initiative.

### Meeting sessions

The workshop was organized in four main plenary sessions. The first three sessions comprised presentations by malaria experts followed by discussions with all participants based on challenges identified:

- Session 1: Resistance to artemisinin and ACT in the Greater Mekong Sub-Region and Africa: current and future approaches.
- Session 2: Access to treatment for most at risk and hard to reach population and the need for evaluation of malaria interventions.
- Session 3: Insecticide resistance, residual and outdoors transmission: which new strategies and interventions to overcome these challenges?

The last session was an open debate among all participants on how to improve interactions between research institutions, NMCPs, CSOs and financial and technical partners to accelerate translation of research into policies and programmes.

## Session 1: Resistance to artemisinin and ACT in the Greater Mekong Sub-Region and Africa: current and future approaches

The emergence of resistance to anti-malarial drugs [chloroquine (CQ), sulfadoxine-pyrimethamine (SP) and mefloquine (MQ)] occurred historically in western Cambodia, at the border with Thailand. The resistance then spread westward to reach India in the 1970s and East Africa in the 1980s to then extend to the whole African continent by 1992 [2]. This situation led to millions of deaths [3, 4]. A video developed by WWARN [5] shows the spread of CQ and SP resistance from the Thai-Cambodia border to the rest of the world between 1960 and 2006. Anti-malarial drug resistance, as defined by WHO [6] was often documented decades after the events of emergence or spread, leading to a gap between the information gathered by researchers and actions taken by programme managers and implementers. When revisiting the history of CQ and SP resistance, it is clear that the response was slow (delay in changing drug regimen) and that there was an inadequate assessment of the risk and of the cost of resistance. The adoption and use of ACT led to the expectation that control and elimination of malaria could be achieved in a relatively shorter period of time. Unfortunately the emergence of artemisinin and insecticide resistance renders this optimistic scenario unlikely (See [7] for definition of artemisinin resistance). The adoption of artemisinin derivatives in combination with a partner drug as first-line treatment and of artesunate in replacement of quinine for severe malaria reversed this situation for about a decade; until studies showed an increase of parasite clearance time in western Cambodia [8]. The Emergency Response to Artemisinin Resistance (ERAR) framework was released in April 2013 [9] and followed by the establishment of the Resistance Artemisinin Initiative (RAI) and of the WHO ERAR regional hub supported by the Global Fund to fight AIDS, TB and Malaria (GFATM) and by the Bill and Melinda Gates Foundation (BMGF). A multi donor trust fund supported by the Asian Development Bank (ADB) was also recently established to support the response to this issue. Despite this, artemisinin resistance threatens to follow the same historical trajectory from Southeast Asia to the Indian subcontinent as seen in the past with other anti-malarial medicines and further spread or emerge in Africa [10]. In place where high resistance to artemisinin has been observed, failures to a conventional treatment regimen with ACT have been reported [11–16].

*Nicholas White* (Mahidol-Oxford Tropical Medicine Research Unit, Thailand) reviewed the situation of anti-malarial drug resistance in South East Asia (SEA). The Tracking Resistance to Artemisinin Collaboration (TRAC) study conducted in South-East Asia and 3 countries in Africa, between May 2011 and April 2013, has documented slow parasite clearance time [7] in southern Vietnam, central Myanmar, western and north-eastern Cambodia, northern, southern and western Thailand [17]. It is now clear that the artemisinin resistance spread or emerged de novo in other parts of the GMS, and that there are occurrences of multiple independent emergences of resistance [18]. The discovery of the *kelch*-*13* molecular marker associated with delayed parasite clearance time [19] (some mutations beyond the amino acid position 440 being strongly associated with slow parasite clearance) allowed to refine those results by confirming that resistance is now widespread in most of the mainland of South-East Asia, the western front being in Myanmar [17]. In a recent report, resistance to the artemisinin has been reported within 25 km of the Indian border [10].

The *kelch* mutations associated with resistance have only been selected in this region, and there is no evidence for selection of those mutations in other parts of the world. The genetic background, allowing for these mutations to emerge and to persist, is only present in the GMS, leading to believe that we could stop its spread. Transfection studies show that when inserting the mutant gene in a parasite, particularly of Asian origin, reduced susceptibility to artemisinin is induced. But those parasites are not selected elsewhere, suggesting a multigenic basis for resistance. Although mutations beyond amino acid position 440 are associated with resistance, it is not always the case as shown with the mutation in position 578 seen in different locations outside of SEA but not associated with artemisinin resistance.

*François Nosten* (Shoklo Malaria Research Unit, Thailand) discussed the challenges faced for malaria elimination in the GMS. Challenges for elimination of *Plasmodium vivax* were highlighted: hypnozoites and its large number of carriers; the often unrecognized severity and mortality of *P. vivax* infections; the emergence of *P. vivax* resistance to CQ; G6PD deficiency and the risk of haemolysis after treatment by primaquine; and sub-microscopic infections. In line with those challenges, the aim of a project supported by the 5% Initiative in the GMS and involving other institutions (Institute Pasteur of Cambodia, Wellcome Trust units in Thailand, Laos and Vietnam) is to support national programmes to map genetic mutations causing G6PD deficiency as well as setting new assays to better detect gametocytes carriers.

In an on-going project supported by GFATM and BMGF, where 800 villages in Myanmar have been mapped and qPCR surveys conducted in 15 of them, preliminary results show that more than 40% of the population are asymptomatic carriers and about 80% of those with *Plasmodium falciparum* carry the *k13* mutations conferring resistance, probably contributing to the on-going transmission of those artemisinin resistant parasites. The speed of the spread of artemisinin resistance westward in Myanmar is alarming: a retrospective analysis of blood samples collected on the Thai-Myanmar border since 2003 shows that the K13 molecular marker proportion in the population has increased. The number of cases has declined and as a result the proportion of K13 mutants has increased, but the high proportion (80%) of K13 mutants is also found in the sub-microscopic reservoirs, hence the urgency to eliminate them as fast as possible, because the parasites that are now transmitted are almost all resistant (Unpublished T. Anderson ASTMH 2014).

Preliminary results from studies (supported by BMGF and Wellcome Trust) conducted in villages at the Thai-Myanmar border, as well as in Cambodia and Vietnam, show that following Mass Drug Administration (MDA) monthly for 3 months, elimination of asymptomatic carriers seem feasible in some villages after 18 months, although in other villages, malaria symptomatic and asymptomatic cases persist after 18 months. This difference in outcomes is likely due to low level of community participation and population movements. Following a question on the risk of increasing drug pressure on the parasite when using MDA, it was further clarified that as asymptomatic carriers have acquired some immunity and have very low parasitaemia, the probability of selection of resistance is very low.

*Véronique Sinou* (Aix-Marseille Université, France) presented the advantages of an ultramobile laboratory (K-LMP) for the surveillance of malaria in Democratic Republic of Congo (DRC). This project is supported by the 5% Initiative and involved several institutions (Aix-Marseille University, France; Monkole Medical Centre, DRC; Centre de Formation et d’Appui Sanitaire, DRC; National Institute of Biomedical Research, DRC). This project aims to evaluate the susceptibility of *P. falciparum* clinical isolates to eight anti-malarial drugs by associating the ex vivo assay and the analysis of molecular markers of resistance, in the provinces of Bas-Congo and Kinshasa. The K-LMP is made of four compartmented boxes stackable by pairs. In its operational configuration, the boxes 1 and 4 (88 × 64 × 100 cm; 0.45 m^3^) rest on the boxes 2 and 3 (88 × 64 × 72 cm; 0.35 m^3^), respectively. These are easy to transport from one study site to another allowing to bring closer to the patients the means necessary to the collection and processing of the samples, for the diagnosis, and to the achievement of ex vivo assays with the same quality as in reference laboratories, especially regarding the reproducibility of in vitro culture parameters [20]. To do this, the boxes are equipped with a class II biological safety cabinet and an incubator adapted to field conditions, and a field suitable ELISA reader. This laboratory environment can be set up in 30 min without specific tools and does not need qualified technicians.

Preliminary results from ex vivo studies showed resistance to chloroquine and reduced susceptibility to quinine, as well as to mefloquine in the two provinces; isolates were susceptible to lumefantrine and piperaquine. However, isolates from Bas Congo showed reduced susceptibility to amodiaquine (AQ), which could be explained by the proximity with Angola where a genotype (SVMNT on *Pfcrt* gene), which might be linked to AQ resistance, has been recently identified. Molecular marker analysis might help to identify if this genotype is present in this region of DRC. In addition, one isolate from Bas Congo presented a reduced susceptibility to dihydroartemisinin.

The finding of varying level of parasites susceptibility at two sites is not surprising in a large country such as RDC, but emphasizes the need for systems for the monitoring of parasite resistance so as to best adapt the treatments to the local realities. For this, training and capacity building of laboratory techniques and methodology are provided to partners in DRC with the aim to have the medical and laboratory staff to be autonomous for conducting field studies by this end of the project.

*Philippe Guérin* (WWARN) gave a presentation on: “Risk of anti-malarial drug resistance worldwide: are we ready?” The fact was emphasized that from an epidemiological point of view, the current situation is an emergence and an emergency, and that artemisinin resistance should be considered as such.

*Intelligence and clinical trials* The WWARN data centre on clinical assessments currently contains over 110,000 individual patient data, representing more than 2/3 of all ACT published data, collected between 1995 to date. Overall this represents a large number of studies, however when looking at the last decade, relatively much less studies have been conducted, compared to the period 2000–2005 when a lot of attention was given at documenting CQ and SP resistance. Following the introduction of ACT at large scale, the attention of donors and researchers shifted to other issues and much less clinical trials have been conducted especially in Africa.

*Intelligence and molecular markers* A pooled analysis of studies conducted with artemether-lumefantrine showed that the presence of parasites carrying the *pfmdr1* N86 molecular marker is associated with a decrease of clinical efficacy, mostly due to lumefantrine resistance; a similar association of parasites with the *pfmdr1* 86Y marker and poor response to amodiaquine has also been identified [21]. When artemisinin efficacy is compromised, the partner drugs in the ACT are then under very strong selection. Monitoring key molecular markers in *pfmdr1* and *pfcrt* markers could be used to predict lumefantrine or amodiaquine or mefloquine failure of the artemisinin-based combinations that carry those partners. Since artemisinin resistance is widespread in the GMS, this situation is common, and has led already to very significant failure of DHA-piperaquine in Western Cambodia [14, 16, 22, 23].

Similarly in the GMS, the proportion of parasites with a *k13* mutant could be used to monitor parasites that clear slowly after artemisinin treatment [19]. In 2013–2014, blood samples were collected in about 50 sites in Myanmar, showing the extension of resistance up to the Indian border in the northern part of the country, and studies on the Myanmar/China border have also shown high levels of *k13* mutant parasites with slow clearance [10, 24, 25].

*Intelligence and anti-malarial quality* Anti-malarial quality characteristics can be summarized as: falsified, substandard or degraded. These characteristics lead to sub-optimal drug exposure and subsequently to: death and disability, economic losses, loss of faith in health systems and drug resistance [26]. In order to understand the existing data, WWARN developed a comprehensive, open-access, global database that collates customized summaries of all published anti-malarial quality reports since 1946 [27]. No publicly available reports on the quality of anti-malarials are available for 60.6% (63) of the 104 malaria-endemic countries. Of 9,348 anti-malarials sampled, 30.1% (2,813) failed chemical/packaging quality tests. Most reports of this review did not distinguish between falsified, sub-standard or degraded medicines.

A recent study [28] shows that, based on a simulation where 30% of artemisinin-based combinations failed and treatment to severe malaria had to revert to quinine, each year would experience an increase of more than 116,000 deaths due to malaria, an excess of 32MUS$ in health care costs and more than 385MUS$ productivity loss due to extended patient illness. A lot of research is done, but more is needed to effectively translate research results into public health action by:

The main recommendations from session 1 were:

- Given that the spread of artemisinin resistant parasites to India could be the first step in their spread to Africa; the current priority must be to address this problem in South-East Asia before it can become a problem in Africa. To avoid future malaria epidemics in all areas, it is crucial to act now to eliminate artemisinin resistant parasites in the GMS before the ACT completely loses its efficacy.
- Although *P. falciparum* elimination in the GMS is realistic, feasible and particularly urgent in the context of drug resistance, the main challenges are to ensure community participation, to address residual vectorial capacity. Operational research is crucial to address those challenges innovatively.
- Engaging political support and cooperation at all levels, from donor institutions, governments, national programmes and the WHO is intensely needed. Without this comprehensive commitment elimination will not be possible.
- Information must be gathered on the response of parasites where elimination is planned, and the analysis and proper interpretation must be provided to guide the appropriate action in each case. However, crucial intelligence gaps persist, and these must be filled urgently.
- Given the extreme risk to ACT efficacy currently seen in the GMS, preserving ACT potency as long as possible is a paramount priority. When new drugs are introduced, their dosing regimens and surveillance for any signs of resistance must be planned before deployment and rigorously pursued to sustain their efficacy for as long as possible.

## Session 2: Access to treatment for most at risk and hard to reach population and the need for evaluation of malaria interventions

Population movement and migration from areas of drug resistance contribute to the potential spread of drug resistant parasites, it also potentially contributes to reintroduction of the parasite in areas where the disease has been eliminated or is not anymore a public health problem. Due to their mobility patterns, the remoteness of their location or the illegal aspects of their activity, these mobile and migrant populations, are often hard to reach with current malaria tools and interventions [29–31].

Due to the ecology of vectors, malaria in SEA is a problem of the forest and, therefore, of border areas in this region. In this context, population at risk are people living in or close to the forest, local population often ethnic minorities and mobile and migrant population looking for work or new patches of land. The main activities MMPs are involved in are: farming; employed work on plantations, on construction sites (dams, roads), mines; uniformed personnel (border control); forest products collection. Different groups with different activities have different level of malaria risk, mainly biological and socio-economic. The biological risk depends on the level of immunity, on timing, duration and frequency of interactions with the forest. The socio-economic risk depends on poverty and knowledge about malaria (prevention, recognition, treatment). Newcomers, coming from areas with no malaria, have low knowledge of malaria and therefore might not seek appropriate diagnosis and treatment. As they are new in a community they may have no knowledge of health services and may not be known by health services. In addition, some of them might not be willing to be reached in the case of illegal activities (logging).

*Shunmay Yeung* (London School of Hygiene and Tropical Medicine) presented on the issue of access to hard to reach and most at risk population, i.e. Mobile and Migrant Population (MMP), focusing on the situation in Cambodia. The MMP is heterogeneous and includes different groups with different activities and related risks, and, therefore, requiring different approaches for malaria control and elimination. In order to identify the most appropriate interventions and to define and quantify the malaria risk for distinct groups of MMP in Cambodia, a population movement framework (PMF) was developed to inform the National Strategy. The main challenges include: the difficulties to access the most mobile, highest risk populations, especially those in remote areas and engaged in illegal activities; the limitations of current vector control interventions; and the quality of, and adherence to ACT and availability of artemisinin monotherapies in the private sector.

In order to address those challenges, some new initiatives have taken place in Cambodia in different strategic areas: Behaviour change communication (BCC), for example billboards on roads between urban areas and forest areas and the use of taxi drivers as health communication agent; Prevention, for example the piloting of insecticide treated hammock nets (ITHN), impregnated clothing, personal and spatial repellents, and a “forest package”, including ITHN and repellent; Case Management including the social marketing of RDTs and ACT through private providers and the expansion of the network of village malaria workers (VMW) in static villages to mobile malaria workers (MMW) and plantation malaria workers (PMW); Cross-border check points, showing that a high proportion of K13 mutation carriers were observed at the Cambodia-Lao border; and Surveillance including the piloting for MMWs and PMWs collecting information on malaria among MMP and to try to track them down.

Most of these initiatives have been relatively small scale pilot projects with differences in objectives, description and measurement of outcomes making it difficult to make robust conclusions about which interventions should be scaled up and how. Further operational research would, therefore, be useful to document the risk of malaria in different types of MMP (as was recently done in a large survey of rubber plantations), and to explore different approaches (including community led approaches) targeting the MMP ensuring careful documentation of the effectiveness of the interventions and lessons learnt in terms of feasibility for scale-up.

*Arjen Dondorp* (Mahidol-Oxford Tropical Medicine Research Unit) reviewed the GFATM RAI grant providing an overview of the current status of implementation. The RAI is a regional grant of 100 MUS$ allocated to five countries in the GMS (Cambodia, Vietnam, Lao PDR, Myanmar and Thailand), which aims to tackle the problem of *P. falciparum* artemisinin resistance. Myanmar with the highest malaria burden and most people at risk in the region is receiving the biggest part of the grant (40M$), 15 M$ are allocated for the inter-country component (ICC) interventions, especially for border areas where hard to reach population might not be covered by national malaria programmes.

The main oversight body of the RAI is the Regional Steering Committee (RSC), which is driven by performance and impact. The RSC main responsibilities are: (1) ‘high-level’ oversight of RAI project implementation (2) taking collective responsibility for strategic direction, and (3) allocating and reallocating GFATM resources as needed and/or when new evidence becomes available. The RSC is composed of voting and non-voting members as well as observers ensuring a broad representation of all stakeholders.

The ICC has been developed by the RSC (with financial support and technical expertise from the 5% Initiative) with a focus on border population using a “3 M” approach: mapping of hotspots (Map); Eliminate malaria in hotspots (Mop) including establishment VHW network and presumptive treatment of high risk population; Monitoring impact and document changes (Monitor). WHO Malaria Policy Advisory Committee (MPAC) has adopted an elimination agenda for the GMS, which should be then formalized into a WHO policy and national strategies.

Recommendations from an advisory report to MPAC on “Feasibility of *Plasmodium falciparum* elimination in the GMS: technical, operational and financial challenges” are already being used by the RSC for reprogramming of the year 2 of the RAI grant [32].

The WHO-ERAR hub has planned a number of activities focusing on MMP and border or cross border activities which should be funded through the RAI and will work closely with the GMS countries to formulate their elimination action plans and priorities.

*Sébastien Boyer* (Institut Pasteur, Madagascar) presented a multidisciplinary approach to evaluate the effectiveness of malaria interventions, the PALEVALUT project supported by the 5% Initiative [33]. He noted that numerous and various control methods are used in different countries and that usually more than one is being used in a given country. This overlap of interventions in space and time makes it difficult to identify a causal relationship when programme objectives or impact are not achieved.

The objective of the project is to define performance indicators (PI) related to simple questions of effectiveness of interventions: Is it working as expected? PI: protective effectiveness, bio-efficacy; is it carried out as expected? PI: Coverage, management; what hinders effectiveness? PI: Determinants; Do we get what we pay for? PI: Cost effectiveness.

A toolbox has been developed based on existing tools from various disciplines: epidemiology, socio-anthropology, health economy, biology, entomology, parasitology. The use of this toolbox was presented: tools and methodologies from each discipline were used and applied on a given population combining cross-sectional survey, household qualitative survey, case–control study, stakeholders interview and entomological investigations. Performance indicators and indicators of effectiveness were derived from those surveys in an integrated way.

As an illustration of this approach, preliminary results were presented on the effectiveness of IRS: the epidemiological component showed, in a sample of 2046 individuals, no association between IRS and *Plasmodium* infection. It was observed, through household quantitative questionnaires, that in houses were IRS was conducted, ITNs use was lower, leading to think, in first analysis, that people felt protected by IRS and did not need ITNs. However, qualitative household interviews showed that people did not feel protected by IRS, and that IRS was perceived at reducing nuisance (fleas, cockroaches) but not at reducing mosquitoes or not aimed at malaria. Additionally, entomological investigations showed that only a few percents of households met WHO standards of persistence of insecticide activity after 3 months.

To ensure operability, the project is build in three phases of evaluation of the Standard Operating Procedures, each following an implementation phase in countries with different settings [two countries in 2014 (Madagascar and Bénin) and three more countries in 2015 (Côte d’Ivoire, Cameroon and Niger)]. The main objective is to see which tools can be standardized, the ultimate goal being to evaluate a given control method for less than 200.000US$ in 1 year.

The main recommendations from session 2 were:

- Operational research and new ways of evaluation are needed for MMP interventions, as routine health information systems have limitations and might not allow to capture the information needed, and existing type of surveys might not be sufficient for monitoring interventions for MMP. Examples of new approaches used for surveying the MMP recently are: respondent driven sampling, cross-border surveys, plantation surveys.
- Focusing on MMP is a priority given that they are at high risk of malaria and contributes to the spread of artemisinin resistance, however, more operational research (OR) is needed on documenting the malaria risk among different types of MMP, innovative tools and interventions as well as designing implementation in a way that can be evaluated, lessons learned and programmes adapted in an ongoing process.
- Addressing the issue of artemisinin resistance in the GMS requires a sense of urgency, a common spirit, good coordination, regionally adapted integrated strategies, impact evaluation and good surveillance, collaboration between public and private sector, targeting difficult to reach populations, targeting the asymptomatic reservoir, adequate funding, and persistence till the end goal is reached.
- Multi-disciplinary approaches should be further explored as the combination of methods used in synergy provides a better understanding of the determinants of effectiveness of malaria interventions than each of this method alone.

## Session 3: Insecticide resistance, residual and outdoor transmission: which new strategies and interventions to overcome these challenges?

Residual and outdoor transmission, falling out of reach of effective and sustainable prevention measures, particularly for mobile people, constitute another emerging challenge for which new tools and strategies are urgently needed, both in Africa and in Asia [34, 35]. Residual transmission is defined as the malaria transmission remaining when there is good coverage with effective LLINs or IRS interventions. However, given the nocturnal biting behaviour of malaria vectors, during the period before sleeping time or early in the morning, there is no protection from mosquito bites. In addition human behaviours, especially activities conducted outside during night time, further limit protection [34]

Malaria vectors have heterogeneous behaviours (early biting or outdoor versus indoor biting) varying by species, but not only, as some species may have a different biting behaviour depending on the geographical location, all those variations illustrating behavioural plasticity [36, 37]. Mosquito behaviours and population might be selected over the long term by interventions/vector control measures, leading to: (1) a shift of species, as documented in Kenya where increasing coverage of LLINs led to an increasing proportion of *Anopheles arabiensis*, replacing progressively *Anopheles gambiae* [38]; (2) a shift to early biting, as in Benin, where scale-up of LLINs to universal coverage and replacement of LLINs led to selection of early biting mosquitoes [39]; (3) a shift to outdoor and early biting, as in Solomon islands, where after use of IRS with DDT, the relative proportion of *Anopheles farauti* with outdoor and early biting behaviour has increased [40].

Mosquito resistance to at least one insecticide has been identified in at least 64 malaria-endemic countries worldwide and it continues to rise in Africa, South East Asia and Latin America [41, 42]. A Global Plan for Insecticide Resistance Management (GPIRM) in malaria vectors has been developed by WHO and RBM in 2012 to contain this threat [43]. In SEA, most of information on IR comes from the MALVECASIA network set up between 2002 and 2005. It was found that IR was present and that there were several foci of resistance: DDT resistance in *Anopheles dirus* was suspected in Cambodia; resistance to pyrethroid of *Anopheles minimus* in Vietnam; or of *Anopheles epiroticus* in southern Vietnam [44]. The *kdr* mutations, that confer resistance to pyrethroids and DDT were also found, in different vectors, including *Anopheles sinensis* [45]. According to WHO there were no reports of insecticide resistance in Thailand and Lao PDR [46], however the question was whether there was no resistance at all or that resistance was not (or under) reported.

*Marc Coosemans* (Institute of Tropical Medicine, Antwerp) reviewed the current status on residual transmission and the challenges it represents for malaria elimination. Whether the selection pressure on behavioural traits will lead to behavioural resistance and lead to a rebound of residual transmission remains a question. However, the emergence and the selection of heritably altered behaviour traits would be very complex to demonstrate. Regardless of whether this residual transmission is due to pre-existing behavioural resilience or behavioural resistance, new vector tools need to be developed to address the gap not covered by LLIN or IRS interventions [34, 35].

Various vector control initiatives to address residual transmission are currently in place including: the Grand Challenges of the BMGF supporting “New approaches for addressing outdoor/residual malaria transmission”; the Innovative Vector Control Consortium (IVCC) call for “Responding to the challenge of outdoor transmission of malaria”; the WHO Vector Control Advisory Group (VCAG) evaluating new paradigms and tools; the RBM Vector Control working Group (VCWG) working on how to address outdoor/residual malaria transmission. A guidance note was recently issued by WHO-GMP on “Control of residual parasite transmission” [47].

Some innovative tools to address residual transmission include: topical and spatial repellents, attractive baits and traps. Topical repellents are very effective against mosquito bites, and has been shown in Bolivia to offer personal protection against malaria [48]. High coverage of repellent use can significantly reduce man-vector contact and so the malaria transmission [49, 50]. However, recent studies in Cambodia, Lao [51] and Ethiopia [52] failed to demonstrate community protection when using topical repellent at large scale, mainly because of lack of compliance to use repellent daily and consistently in early evening or morning, even when acceptance of the product was high. People indeed like to use repellents as a comfort tool when facing high mosquito nuisance but not for daily use to prevent a disease. With incomplete coverage and compliance it is likely that mosquito diversion will occur from users to non-users. New paradigms such as spatial repellents, attract-and-killing baits and traps are still in the process of evaluation to raise epidemiological evidence and the public health value of these approaches [53].

*Vincent Corbel* (Institut de Recherche pour le Développement, IRD) reviewed the current status of insecticide resistance (IR) globally and presented the MALVEC project currently implemented in the SEA region through the 5% Initiative. The growing threat of insecticide resistance for malaria control and elimination worldwide was emphasized. The MALVEC project was set up to evaluate IR levels, type and mechanisms in the Lao PDR and the Thai-Lao border through a collaboration between the Institut Pasteur in Lao PDR, the Center for Malarialogy, Parasitology and Entomology in Lao PDR, IRD and Kasetsart University in Thailand. The main objectives of the project are Research, Training and Expertise with four main components: (1) evaluation of anopheles bionomics and distribution and their role in malaria transmission; (2) evaluation of the level, type and mechanisms of resistance to public health pesticides; (3) evaluation of the impact of environmental determinants (agricultural/PH practices) on vector dynamics and resistance selection; (4) strengthening capacity of Lao partners in medical entomology. The project follows the recommendations of the GMAP which calls for member states to implement an active system of insecticide resistance monitoring in vectors in order to improve preventive strategies and the fight against malaria worldwide.

Preliminary surveys conducted in the Vientiane province, Lao PDR (using both human landing catch and cow bait collection) show that among mosquitoes collected during the rainy season (4,032 anopheles) and the dry season (3,132 anopheles) 6% (out of 22 species) and 67% (out of 19 species) were primary vectors (*Anopheles maculatus* and *An. minimus*), respectively. WHO susceptibility tests showed that most of anopheles species were still susceptible to pyrethroids but some species (*An. maculatus*, *Anopheles philippinensis*, *Anopheles vagus*) show incipient resistance to DDT, whereas *An. vagus* showed incipient resistance to pyrethroids. In Thailand, at the border with Lao PDR and Cambodia, among mosquitoes collected during the rainy season (3,775 anopheles) and the dry season (378 anopheles), 1% (out of 13 species) and 27% (out of 11 species) were primary vectors, respectively WHO bioassays showed resistance in all sites in *Anopheles peditaeniatus* and resistance to permethrin was suspected in *Anopheles barbirostris*, a secondary malaria vector. Preliminary findings showed that IR was present in anopheles species among which secondary malaria vectors but not in primary vectors. To conclude, IR is present at low levels in the Lao PDR, but is likely to increase by migration from neighbouring countries where resistance has been detected [44] Resistance in mosquitoes can spread to very long distance through migration [54]. More recently the *kdr* mutations has spread to all Africa mainly by migration events [55, 56]. In contrast occurrence of de novo mutations is rare and does not represent the main cause of resistance diffusion around the world. Resistance can also increase by selection considering the increasing use of insecticides in agriculture and public health (e.g. LLIN coverage).

The main recommendations from session 3 were:

- Universal coverage with LLINs should remain a priority as it suppresses most of the malaria transmission due to primary vectors that feed predominately on humans sleeping inside the houses. This has largely contributed to the achievements in the decline of malaria. However, recognizing the limits of IRS or LLINs there is an urgent need for additional vector control measures for personal and community protection, the latter meaning that non-users would have the benefit of the protection.
- The magnitude of the residual transmission should be evaluated in every country, regarding both mosquito and human behaviours.
- There is a need for industry and academic partners to develop new vector control methods and paradigms for outdoor and residual transmission.
- Regular monitoring of insecticide resistance should be continued in order to prevent further occurrence/development of resistance in malaria vectors in the greater Mekong region. Data should be made available to member states and WHO as part of regional plan for insecticide resistance management.

The final session was an open debate on *How to improve interactions between research institutions, national malaria control programmes and the donor community to accelerate translation of research into policies and programmes?*

The debate was structured around three main interrelated topics and the main recommendations are summarized below.

### Current and potential future approaches to improve interactions between the research community and national programmes and donors

- Data should be shared as early as possible to allow NMCP to use findings to adjust their national strategies.
- In the future, research design and strategy might need to be adapted from its classical approach, in collaboration with implementers, to be closer to a “learning by doing” approach to ensure timely data sharing and strategy adjustment.
- Better coordination between stakeholders (country national programmes, researchers, donors) is needed in order to set up clear priorities at country level.

### Governance structure and strategy needed to address the threat of artemisinin resistance

- More involvement of heads of government is essential to engage in the multi-sectoral approach needed, including non-health ministries.
- India and Bangladesh should be invited to join the RAI oversight committee as observers, similar to China’s position at the moment. The need to find ways to include and engage African countries in this process was as well highlighted.
- Existing structures, like the RAI and RSC should be used and reinforced and the next East Asia summit of heads of states should be used to convey the emergency for deeper commitment and to propose a budgeted plan to address artemisinin resistance in the next few years, in the broader context of the 2030 target.

### Better communication and advocacy needed for better translation of research into policies

- Based on scientific evidence and information available, simple and clear key messages should be “packaged”, expressing a common position, a common voice, from the scientific community. It should address the problem, the needs, the priority actions, a budgeted plan and the risks of “doing something versus doing nothing”.
- Key messages should be disseminated to a broader, non-scientific audience, including grass root communities exposed to and affected by malaria, national malaria control programmes, donors and international institutions and importantly policy makers and governments.

## Conclusions

This meeting report present the outcomes of a 1-day workshop which convened a global community of over 80 researchers, representatives of NMCPs, of CSOs, and of financial and technical partners. More operational research and adapted evaluation methods are needed to better address challenges for malaria control and elimination and will require innovation. Those challenges include: ensuring adequate community participation for new strategies, i.e. MDA and preventions methods for residual and outdoors transmission; closing intelligence gaps regarding surveillance of drug and insecticide resistance, anti-malarial medicine quality; building capacity to better monitor insecticide resistance and mosquito behavioural changes linked to scale-up of vector control interventions.

The threat and the emergency of the artemisinin resistance spread and independent emergence in the GMS was intensely debated as it is now close to the border of India. The need for key messages, based on scientific evidence and information available and disseminated without delay, was highlighted as crucial for an effective and urgent response. Those messages should be disseminated to broader, non-scientific audience, including grass root communities exposed to and affected by malaria, NMCPs, donors and international institutions and importantly policy makers and governments.

## Additional file

Additional file 1. List of participants
