# Supplementary material for: Past and new challenges for malaria control and elimination: the role of operational research for innovation in designing interventions
Source: Malar J. 2015 Jul 17;14:279. doi: 10.1186/s12936-015-0802-4 (PMC4504133; doi:10.1186/s12936-015-0802-4)
Supplement: Additional file 1. — List of participants [file 12936_2015_802_MOESM1_ESM.pdf]

Name	Surname	Organization
Dr. Victor	Kaput Makwenge	Honorary Minister of Health, Democratic Republic of Congo
Prof. Graham	Brown	Nossal Institute for Global Health, the Univeristy of Melbourne
Dr. Wichai	Satimai	Department of Disease Control, Ministry of Public Health, Thailand
Mr. Alastair	Robb	Department for International Development, UK
Dr. Bernard	Nahlen	President's Malaria Initiative, USAID
Ms. Valentina	Buj	UNICEF
Sanne Henrietta	Fournier - Wendes	UNITAID
Dr. Sébastien	Marcombe	Institut Pasteur du Laos
Dr. Didier	Fontenille	Institut Pasteur du Cambodge
Dr. Didier	Ménard	Institut Pasteur du Cambodge
Prof. François	Nosten	Oxford, Shoklo Malaria Research Unit, Mae Sot, Thailand
Dr. Sébastien	Boyer	Institut Pasteur de Madagascar
Dr. Léonardo	Basco	Institut de Recherche pour le Développement
Dr. Philippe	Guérin	WWARN
Dr. Mehul	Dhorda	WWARN
Mr. Jeffery	Smith	WWARN
Dr. Jean	Gaudart	Aix-Marseille-Université
Dr. Thomas	Teuscher	Roll Back Malaria
Jacques	Berger	Institut de Recherche pour le Développement
Nadine	Fievet	Institut de Recherche pour le Développement
Dr. Bouasy	Hongvanhthong	Centre for Malaria Parasitology and Entomology, Lao PDR
Dr. Siv	Sovannaroth	National Malaria Center, Cambodia
Dr. Aung	Thi	Department of Health, Ministry of Public Health, Myanmar
Dr. Jetsumon	Sattabongkot Prachumsri	Faculty of Tropical Medicine Mahidol University, Bangkok, Thailand

Dr. Vincent	Corbel	Institut de Recherche pour le Développement
Véronique	Sinou	Université Aix-Marseille
Dr. Fatoumata	Nafo-Traore	Roll Back Malaria
Prof. Marc	Coosemans	Institute of Tropical Medicine, Antwerp, Belgium.
Prof. Nick	White	Mahidol-Oxford Tropical Medicine Research Unit
Arjen	Dondorp	Mahidol-Oxford Tropical Medicine Research Unit
Dr. Shunmay	Yeung	London School of Hygiene and Tropical Medicine
Dr. Deyer	Gopinath	WHO Thailand
Natakorn	Jittanonta	Malaria Consortium Thailand
Rush	Ashton	Malaria Consortium Cambodia
Dr. Hnin Su Su	Khin	PSI Myanmar
Ms. Abigail	Pratt	PSI Myanmar
Dr. Myo Kyaw	Lwin	Save the Children Myanmar
Attila	Molnar	UNOPS Myanmar
Mr. Michael	O'Dwyer	AUSAID
Ben	Rolfe	Asia Pacific Leaders Malaria Alliance
Dr. Sandii	Lwin	Myanmar Health and Development Consortium
Amélie	Joubert	RAI Regional Steering Committee, WHO
Ms. Izaskun	Gaviria	The Global Fund
Dr. Sally	Stansfield	Deloitte Consulting
Dr. Vanessa	Racloz	Global Malaria Action Plan, WHO - GMAP 2
Dr. Helen	Prytherch	Swiss Tropical and Public Health Institute - GMAP2
Dr. Nicolaus	Lorenz	Swiss Tropical Institute
Dr. François	Desbrandes	Sanofi Aventis
André	Tchouatieu	Sanofi Aventis
Dr. Louis	Da Gama	Global Health Advocates

Dr. Promboon	Panitchpakdi	Raks Thai Foundation
Nou	Sanann	FHI 360
Dr. Lorenz	Von Seidlein	Mahidol-Oxford Tropical Medicine Research Unit
Bob	Taylor	Mahidol-Oxford Tropical Medicine Research Unit
Dr. Hans	Hovergaard	Norwegian University of Life Sciences
Prof. Srivicha	Krudsood	Faculty of Tropical Medicine, Mahidol University
Khun Anusorn	Pawaputanun	Ministry of Public Health, Thailand
Dr. Anupong	Sujariyakul	Ministry of Public Health, Thailand
Dr. Hubert	Barennes	Institut Pasteur du Cambodge
Prom	Chanrith	KHANA
Dr. Joel	Tarning	Mahidol Oxford Tropical Medicine Research Unit, Faculty of Tropical Medicine, Mahidol
Dr. Jeeraphat	Sirichaisinthop	Department of Disease Control, Ministry of Public Health, Thaildand
Le Thi Kim	Chi	RESED CENTER
Dr. Oahn	Khuat	Center for Supporting Community Development Initiatives (SCDI)
Rima	Shretta	Malaria Elimination Initiative<br>Global Health Group, University of California, San Francisco
Prof. Maxine	Whittaker	Asia Pacific Malaria Elimination Network
Adeline	Lautissier	Initiative 5% - France Expertise Internationale
Clarisse	Veylon	Initiative 5% - France Expertise Internationale
Audrey	Giret	Initiative 5% - France Expertise Internationale
Néra	Khamvongsa	Initiative 5% - France Expertise Internationale
Philippe	Guyant	Initiative 5% - France Expertise Internationale
Mathilde	De Calan	Ministère des Affaires étrangères et du Développement international
H.E. Thierry	Viteau	Ambassade de France en Thaïlande
Eric	Fleutelot	Ambassade de France en Thaïlande
H.E. Philippe	MEUNIER	Ministère des Affaires étrangères et du Développement international
